# Laser Bioprinting of Cells Using UV and Visible Wavelengths: A Comparative DNA Damage Study

**DOI:** 10.3390/bioengineering9080378

**Published:** 2022-08-09

**Authors:** Panagiotis Karakaidos, Christina Kryou, Nikiana Simigdala, Apostolos Klinakis, Ioanna Zergioti

**Affiliations:** 1 Biomedical Research Foundation, Academy of Athens, 11527 Athens, Greece; 2 School of Applied Mathematical and Physical Sciences, National Technical University of Athens, 15780 Zografou, Greece; 3 Institute of Communication and Computer Systems, 15780 Zografou, Greece

**Keywords:** laser-induced forward transfer (LIFT), wavelength, laser fluence, DNA damage, double strand breaks

## Abstract

Laser-based techniques for printing cells onto different substrates with high precision and resolution present unique opportunities for contributing to a wide range of biomedical applications, including tissue engineering. In this study, laser-induced forward transfer (LIFT) printing was employed to rapidly and accurately deposit patterns of cancer cells in a non-contact manner, using two different wavelengths, 532 and 355 nm. To evaluate the effect of LIFT on the printed cells, their growth and DNA damage profiles were assessed and evaluated quantitatively over several days. The damaging effect of LIFT-printing was thoroughly investigated, for the first time at a single cell level, by counting individual double strand breaks (DSB). Overall, we found that LIFT was able to safely print patterns of breast cancer cells with high viability with little or no heat or shear damage to the cells, as indicated by unperturbed growth and negligible gross DNA damage.

## 1. Introduction

The ability to precisely position cells is essential for establishing cellular architecture and creating the organ structures needed to understand cellular interactions in normal or diseased tissues and to make functional tissue replacements. Therefore, bioprinting technologies have been employed to study spatially controlled cell–cell and cell–environment interactions [1,2,3]. Currently, various bioprinting techniques are used, including drop-on-demand techniques, such as inkjet printing [4], laser-based printing (also called laser-induced forward transfer, LIFT) [5,6] and extrusion printing [7]. Among these different bioprinting technologies, LIFT, a non-contact printing technique, possesses the ability to print various biomaterials with high precision, including proteins [8], DNA [9], living cells [10,11,12,13,14,15], and cell-encapsulating hydrogels [16,17,18]. On the other hand, orifice-based techniques, such as inkjet printing, have inherent limitations, including nozzle clogging and high shear force at the nozzle, which eventually lead to impaired cell survival [4,19,20]. As a result, only biomaterials with low viscosity and cell suspensions with low density can be printed. Since LIFT-printing is a nozzle-free printing technique, it is advantageous for the direct writing of viscous materials (1–300 mPa/s) [21], or dense cell suspensions up to 1 × 10^8^ cells/mL, with high resolution [7]. LIFT utilizes laser transparent donor substrates, coated with a thin layer of metal or polymeric laser-absorbing material, on which the bio-ink is loaded and printed onto a receiver substrate lying opposite. LIFT can transfer hydrogel biomaterials, biomolecules and living mammalian cells at a 10 to 100 μm scale [22].

The goal of bioprinting is to create functional 3D structures that mimic native tissue architecture and function. The immobilization of the printed cells at desired positions on the substrate enables unique applications compared to other printing/seeding methods, such as the sequential printing of different cell types or ECM components that could closely mimic the in vivo microenvironment. Such 2 or 3D structures could be used in cell biology studies, cancer biology, preclinical testing and, more importantly, in regenerative medicine. A significant challenge, however, is to provide the appropriate microenvironment that the cells need to survive and remain functional following printing. For example, it has been demonstrated that the physicochemical properties of cells in suspension will be slightly changed during printing [23].

Moreover, it is possible that laser-based techniques may introduce thermal and/or mechanical stresses to living cells during laser transfer [24,25]. If this laser-induced stress exceeds the adaptive capacity of a cell, irreversible damage may occur. Cell damage can be simply classified as thermal and/or mechanical cell injury and biochemical injury [26]. In general, cell damage is reversible, up to a certain point, depending on the type and load of damage that a certain cell type can anticipate and/or repair; however, the exposure of cells to high external stress may cause irreversible cell injury and even cell death. Indeed, cell death due to process-induced cell injury is common in laser-based techniques, and cell viability after printing is a key criterion in evaluating the feasibility and efficiency of any laser-based technique [27]. Several studies have been performed to investigate the cell damage caused by LIFT-printing, where laser wavelengths ranging from 193 to 1064 nm [28,29,30,31,32,33,34] and various pulse durations [35,36] were investigated. For LIFT bioprinting, the most used laser pulse duration is the nanosecond pulse [8,37,38,39]. However, the thermal effects of a nanosecond laser can cause damage to heat-sensitive cells during printing [40]. Short wavelength lasers can induce DNA damage and cause photochemical cross-linking in the cell suspension. As a result, cells may suffer gross genomic instability leading to cell death (apoptosis) or carcinogenesis [34]. It is commonly suggested that lasers with shorter pulse duration, such as femtosecond or picosecond lasers, could reduce the heat released to the cell suspension, thus resolving the problem of thermal damage of cells during printing. Among the different types of wavelengths used, infrared lasers (IR) are recommended because they cause less damage to cells than UV lasers [38,41,42]. Nevertheless, previous studies have revealed that LIFT-printing using UV wavelengths caused minor damage to the printed cells even in the absence of a laser-absorbing layer, where 99% of the laser energy passes through the cell suspension [10]. Direct exposure to lower wavelengths (~250–300 nm) without printing can cause serious damage to the cells [43,44]. To date, several studies have shown that a variety of mammalian cell types can be laser-printed without acquiring DNA damage or compromising cell viability [10]. In addition, various other cell types, both carcinomatous and normal, show high viability, irrespective of the laser absorbing layer utilized [33,45,46,47]. Following printing, cell viability remains between 80 to 90%, or even close to 100% [20,44,48,49,50]. Moreover, all these studies show that the cells recover and start to proliferate normally within a short period of time. However, the DNA damage detection methods utilized to date suffer from low sensitivity (detecting gross DNA alterations via comet or TUNEL assays) [20,46], are indirect (e.g., viability or markers of induced stress) [10,42,50] or explore in low resolution [51].

The objective of this study was to investigate possible damage induced by LIFT-printing of cells in the UV (355 nm) and visible region (532 nm). To this end, two breast cancer cell lines with low endogenous DNA damage were identified in a screen of several available normal and malignant cell lines (data not shown) and were used in this study. A low endogenous damage burden was considered a prerequisite to increase the sensitivity of our analysis. At first, the study focused on optimizing the printing process for both wavelengths to determine the printing conditions that were to be used in the following experiments. By examining quantitatively the growth and DNA damage profiles of LIFT-printed breast cancer cells, the study demonstrated that the cells were largely unaffected by the LIFT process.

## 2. Materials and Methods

### 2.1. Cells, Culture and Sample Preparation

The two human breast cancer cell lines MDA-MB-468 andMDA-MB-231 used, as well as human embryonic kidney HEK293T cells, were purchased from ATCC (LCG standards, Merck SA, Johannesburg, South Africa) and cultured in Dulbecco’s Modified Eagle Medium (DMEM, high glucose, SH30243.01, Cytiva, Marlborough, MA, USA), supplemented with 10% fetal bovine serum (FBS12A, Capricorn Scientific, Ebsdorfergrund, Germany) and 1% penicillin/streptomycin (15140–122, Gibco, Gibco, Thermo Fisher Scientific, Waltham, MA, USA) in a humidified atmosphere of 5% CO_2_ at 37 °C. The MDA-MB-468-H2B-GFP cells were generated from MDA-MB-468 after stable transfection with a pINDUCER10-H2B-GFP vector and puromycin selection. H2B-GFP was obtained from pBOS-H2B-GFP using Not1 and Acc65I enzymes, followed by fill-in to generate blunt ends. The insert was cloned into a modified pINDUCER10 [52], where the Ubc promoter and the TRE elements were replaced by the EF1 promoter and the tight TRE elements, respectively. HEK293T cells were used to produce the lentiviral particles. MDA-MB-468 cells were infected with the supernatant and selected with puromycin (2.5 μg/mL, sc-108071A, Santa Cruz) to generate a stable cell line. The MDA-MB-468-H2B-GFP cells were sorted to ensure there was no leakiness of GFP expression in the absence of Doxycyclin (Dox, D9891, Sigma Aldrich, St. Louis, MO, USA). Upon Dox administration (500 ng–1 μg/mL), all cells expressed variable levels of H2B-GFP, readily visible after 12–16 h. MDA-MB-468-H2B-GFP behaved as MDA-MB-468 cells or mock transfected cells in terms of morphology and growth rate (data not shown). All cells were, routinely, passaged every 2 or 3 days and tested for mycoplasma. To prepare bio-inks for printing, exponentially growing MDA-MB-468, MDA-MB-468-H2B-GFPor MDA-MD-231 cells were trypsinised (T4049, Merck SA) and viable cells were counted on a hemocytometer using Trypan blue (15250–061, Gibco) exclusion. The cells were then centrifuged at 1500 rpm for 5 min and resuspended at 75 × 10^3^ cells/μL in medium. Resuspended cells were kept on ice until direct printing onto coverslips (autoclaved sterile, 40058-ATO, Lach-Ners.r.o.), normally within one hour. 

After printing, the coverslips were transferred into 24-well plates and 0.5 mL medium was added to each well. For cell growth studies, the medium was changed every other day. The day before imaging, the medium was supplemented with DOX (1 μg/mL) so that, on the following day, only GFP-expressing (i.e., viable) cells were visualized and counted. For cell death assessment, the medium of printed MDA-MB-468 cells was supplemented with 10 μg/mL Hoechst 33258 and 1 μg/mL propidium iodide (PI, 40017, Biotium, Fremont, CA, USA) and the cells incubated at 37 °C for 15 min, at the indicated timepoints, prior to visualization. For the DNA damage evaluation experiments, the printed cells were washed 3 times with 1xPBS (XC-S2066, Biosera) and fixed in chilled 4% paraformaldehyde (PFA, P-6148, Sigma Aldrich) for 2 h on ice at 0, 6 and 24 h post-printing.

### 2.2. Cell Viability Assay

To assess the presence of apoptotic cells after LIFT, the printed cells were incubated with Hoechst 33258 (10 μg/mL) and propidiumiodide (PI, 1 μg/mL, 40017, Biotium) in the culture medium for 15 min at 37 °C (cell culture incubator) as previously described [53]. Immediately after 3 washes with prewarmed PBS the cells were imaged live. 

### 2.3. Immunofluorescence (IF)

Immunofluorescence was performed as previously described [54], with a slight modification: the step of the secondary antibody was omitted since the primary antibody utilized, anti-phospho-H2A.X Antibody (Ser139), clone JBW30, Alexa Fluor^®^ 555 Conjugate (05-636-AF555 Sigma-Aldrich, Germany), was already conjugated with fluorochrome. The antibody was used at 1:200 dilutions.

### 2.4. LIFT Setup and Laser Source

**Figure 1:**
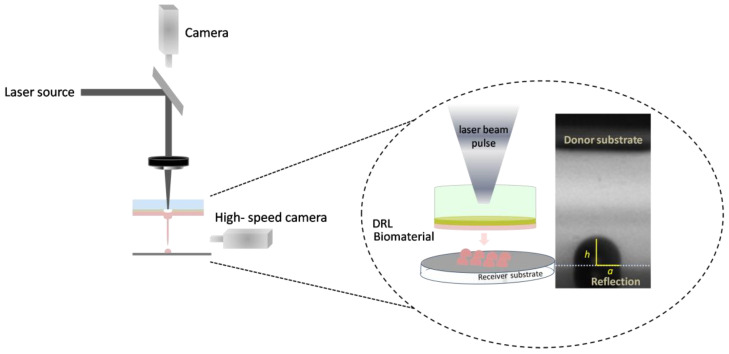
Schematic of LIFT setup.

A schematic representation of the LIFT setup, which was used for the defined deposition of cells, is illustrated in Figure 1. Two different pulsed lasers were used to deposit microarrays of droplets (6 × 6) to study the effect of two different wavelengths on cell viability.

#### 2.4.1. LIFT Setup, at 532 nm (System 1)

The DPSS picosecond STANDA STA-01 laser source employed in this study, operated at a wavelength of 532 nm, and pulse duration of 600 ps at 1 Hz repetition rate. The setup comprised a beam expander, an attenuator plate, a variable circular mask and a plano-convex converging lens (f = 75 mm) and was used to direct and project the laser beam onto the thin laser-absorbing layer. The laser spot size at the donor substrate was fixed at 50 μm and the laser fluence was controlled with the aid of the attenuator. The laser parameters are listed below (Table 1).

#### 2.4.2. LIFT Setup, at 355 nm (System 2)

The nanosecond Nd: YAG laser source (Litron Nano-L 200-10, neodymium-doped yttrium aluminum garnet) employed in this study, operated at a wavelength of 355 nm, with pulse duration of 10 ns at 1 Hz repetition rate. The setup comprised a beam expander, an attenuator plate, a variable circular mask and a plano-convex converging lens (f = 50 mm) and was used to direct and project the laser beam onto the thick laser-absorbing layer. The laser spot size at the donor substrate was fixed at 70 μm and the laser fluence was controlled with the aid of the attenuator. 

Both experimental setups consisted of two positioning systems, namely the donor and the receiver substrate. The donor substrate was a transparent carrier coated with a laser-absorbing layer (also called the dynamic release layer, DRL), onto which the material under transfer (liquid or solid form) was applied, and a receiver substrate which was placed at close proximity and parallel to the donor surface. In brief, laser pulses were focused through the laser-absorbing layer, which was vaporized locally in the focal region of the laser beam. As a result of the laser absorption, a high-pressure bubble forms in liquid and rapidly expands to produce a fast and thin jet with the subsequent separation of one droplet and its transfer to the receiver substrate. Furthermore, the donor-receiver substrates can be moved independently with respect to each other. Thus, any pre-defined pattern and pre-defined object geometries can be fabricated in a layer-by-layer manner. It is important to note that, even if the laser transfer ejection is initiated through a thermal mechanism, only the first few nanometers of the cell suspension on the donor substrate would be volatilized, and the main volume of the cell suspension is transferred with little to no heating [48].

In this study, the donor substrate of “System 1”at 532 nm wavelength, comprised 1-mm-thick glass slides (26 × 76 mm², DELTALAB, Barcelona, Spain) that were cleaned by sequential exposure to isopropyl alcohol, deionized water, and acetone in an ultrasonic bath for 30 s, followed by purging with purified air. The use of absorbing layers of Au has been examined with positive results in the transfer of biomolecules and cells [55]. A volume of 3 μL of cell suspension was applied into the well to obtain an approximately 90 µm thick coating. The cell suspension was printed, in a square array of 6 × 6 droplets with 900 μm spacing between droplets, onto sterilized and gelatin-coated receiving glass coverslips (13 mm in diameter, CCVN-013-100, Labbox, Milan, Italy). In brief, autoclave sterilized coverslips were submerged in ultrapure water with 0.1% gelatin (ES-006-B, Millipore, Merck SA) for at least 30 min in a laminar flow hood. Then, the coverslips were air dried in the hood and used for bioprinting within the day. Primarily, the gelatin layer prevents the printed cells from drying out, but it also cushions the impact of the laser printing process [56]. The laser fluence was varied from 360 to 850 mJ/cm^2^. In the case of “System 2”, at 355 nm wavelength, the donor substrate, 1 mm thick quartz (25 × 25 mm²), was cleaned by sequential exposure to isopropyl alcohol, deionized water, and acetone in an ultrasonic treatment for 30 s, followed by purging with purified air. The laser fluence was systematically adjusted in the range of 330 to 800 mJ/cm^2^. The donor substrate, in both systems, was placed parallel to the receiver substrate, with the liquid film facing the gelatin-coated glass coverslips at a distance of 500 μm and was monitored through a CCD camera. The donor holder was laid on an xyz translation stage that permitted the precise translation of the donor substrate with respect to the laser beam.

### 2.5. Measuring Droplet Diameter, Volume and Jet Velocity

The printed droplet diameters were measured based on the average value of the five representative printed droplets for each case after achieving equilibrium on the receiver substrate. For those droplets followed by secondary droplets, their equivalent feature diameter was estimated based on their equivalent total volume, calculated by assuming a spherical cap segment of all the droplets, using the following Equation (1):

(1) $$V_{\mathrm{cap}}=\frac{1}{6}\pi \times h(3a^{2}+h^{2}),$$

where ‘h’ and ‘a’ represent the height of the jet and the base radius, respectively.

The jet velocity was calculated based on the spatial position difference of the jet front of the two sequential imaging frames of the jetting process from the following Equation (2):

V = (h_2_ − h_1_)/(t_2_ − t_1_), (2)

where ‘h’ and ‘t’ represent the height of the jet and time, respectively.

### 2.6. High-Speed Camera Setup

A side-view imaging configuration was employed by coupling a LIFT setup with a high-speed camera (Mini AX-100, Photron USA, Inc., San Diego, CA, USA, with maximum recording speed at 540 kfps) and a standard LED (Thorlabs LEDD1B Thorlabs GmbH, Lübeck, Germany) placed opposite to the camera for illumination purposes, for the real-time visualization of the ejection process. In this experiment, a recording speed at 127.5 kfps, equivalent to a time resolution of 7.8 µs, was utilized for monitoring the emerging jet. The synchronization of the laser-camera triggering was achieved by a custom-designed LabView program. 

### 2.7. Imaging and Image Analysis

ImageJ software was utilized to process the captured images. IF images of γH2AX immunostained cells were acquired on an upright fluorescence microscope (Leica DMRA2) via Orca Flash 4.0 V3 CMOS Camera (Hamamatsu Photonics, Japan) using a 40× lens. The images were analyzed with the ImageJ software. In brief, DAPI staining was utilized to set the area of interest (ROI), i.e., the entire nucleus of each cell, as well as to automatically measure the number of cells (nuclei) per image. Utilizing the “find maxima” command the γH2AX foci were identified. Out of the total foci per image, those measured were filtered to include only those inside the ROIs of the image. In addition, nuclei that were partially captured at the borders of each image were also excluded from the measurements. Thus, the results output for each image analysis described the number of γH2AX foci per nucleus of that image. Manual inspection was performed in all steps of the image analysis and individually for all acquired images. Cropped and enlarged images of merged pseudocolors (blue and red for DAPI and γH2AX, respectively) were obtained after micro-adjustments to the brightness/contrast of the original images using Adobe Photoshop CS6 software. The same software was utilized to generate the IF panels of relevant figures of the manuscript.

Phase contrast and fluorescent images of LIFT-printed cells (H2B-GFP expressing MDA-MB-468-H2B-GFP cells, as well as MDA-MB-468 cells counterstained with Hoechst 33258 and PI) were acquired live on an inverted phase contrast microscope (Leica DM IRE2) equipped with an Orca Flash 4.0Plus V3 CMOS camera (Hamamatsu Photonics, Japan) using a 5× lens. Hoechst 33258 and PI positive cell counts were performed with the ImageJ software as above for DAPI.

### 2.8. Statistical Analysis

All measurements were analyzed and expressed as mean and standard error of the mean (SEM). For droplet size measurements, at least five droplets per condition were measured. The experiments for DSB and viability measurements were performed in triplicates (three independent experiments).

## 3. Results and Discussion

This study aimed to: (a) investigate and identify the optimal laser parameters of LIFT-printing for the controlled transfer of cell suspensions, which is a major technological challenge for most laser bioprinting techniques, and (b) to thoroughly assess the safety of the printing process in terms of cell viability/growth and putative DNA damage induction.

### 3.1. Optimizing the LIFT Process

**Figure 2:**
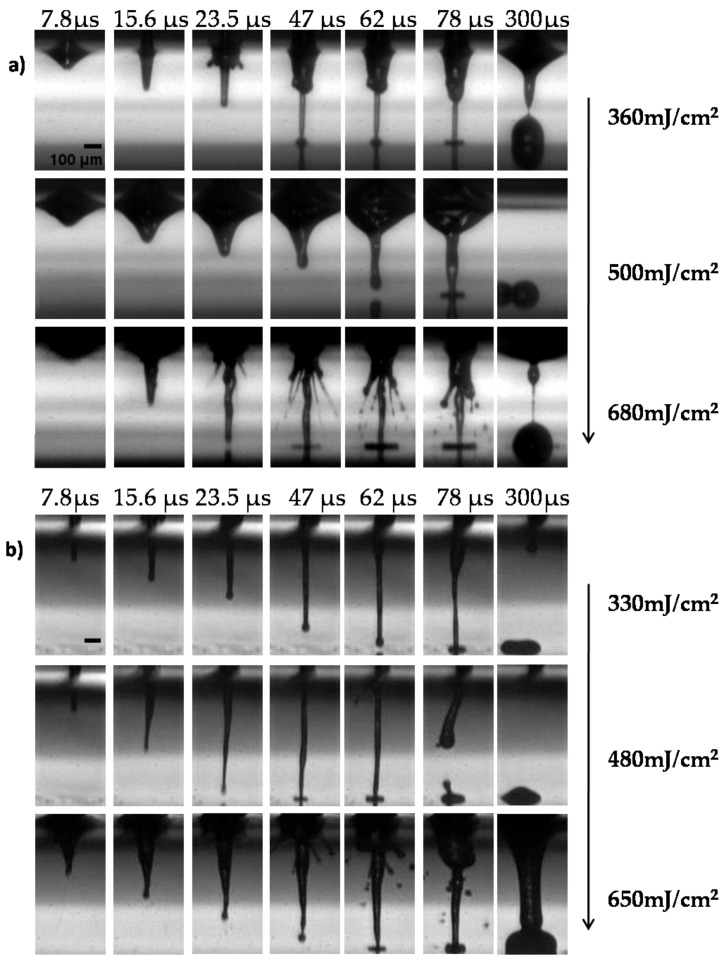
Visualization of the jet dynamics induced by laser pulses with different wavelengths (**a**) at 532 nm and 0.6 ns pulse duration, and (**b**) at 355 nm and 10 ns pulse duration at different laser fluencies at the indicated timeframes. Representative images from each condition are depicted. All experiments were conducted in triplicates, n = 5 Scale bar 100 μm.

The first part of our study aimed to optimize the printing process for both wavelengths via the real-time visualization of cell suspension ejection events. Initially, the effect of the laser wavelength on the printed droplet size was investigated. The laser fluences varied from the minimum needed for cell printing, without satellites, up to laser fluences causing strong splashing of the droplets. Figure 2 depicts selected image frames from the time-resolved imaging experiments, with a defined delay relative to the laser fluence impact for the two different wavelengths with different laser fluences (at ~350 to ~650 mJ/cm^2^).

**Figure 3:**
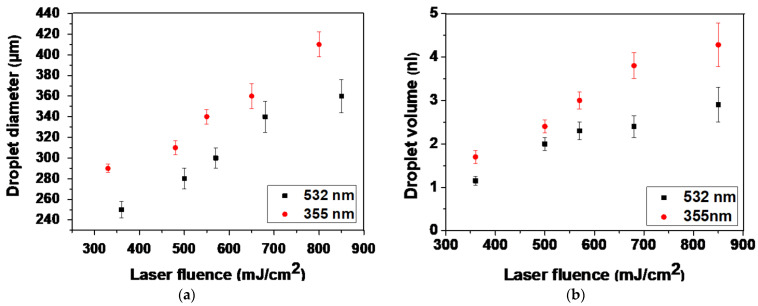
(**a**) LIFT-printed droplet diameter as a function of laser fluence for both wavelengths. (**b**) Droplet volume as a function of laser fluence for both wavelengths. Error bars depict SEM. n ≥ 5 droplets per condition, in at least three independent experiments.

The analysis of LIFT through time-resolved imaging, demonstrated that the formation of stable jets all along their progression away from the donor was followed by well-defined droplets for both lower laser fluences (~350 and ~500 mJ/cm^2^). At lower laser fluences, the droplets preserved their circular shape with little spreading. As the jet progressed and impinged on the receiver substrate, it began to accumulate liquid at the impact position, with practically no deviation from the straight trajectory, maintaining its well-defined shape. In contrast, for higher laser fluences (e.g., at ~650 mJ/cm^2^), the produced jets became unstable and non-directional, while “splashing” behavior was observed. This jetting behavior can be attributed to the overpressure of the generated gas that is entrapped within the cavitation bubble, which can overcome the cohesive forces of the surrounding liquid film and lead to the violent propulsion of both liquid and gas [57]. The images were quite similar for the two wavelengths; at 532 nm, the jet duration was slightly shorter (about 300 µs), while the jet flowed for about 430 µs at 355 nm. In Figure 3a, the relationship between the laser fluence and droplet diameter is illustrated. Above the minimum laser fluence, which was determined at 250 mJ/cm^2^ and 300 mJ/cm^2^ for 355 and 532 nm, respectively (data not shown), the droplet diameter increased almost linearly as the laser fluence increased. When the laser wavelength was varied between the two laser sources for identical laser fluence, there was a significant change in the printed droplet diameter. At 355 nm, lower laser fluence was required to deposit a droplet. The error bars were determined by taking the standard error of the mean (SEM) of the droplet diameter of at least five droplets at the given laser energy from multiple experimental runs. The ImageJ software was utilized to process the captured images as well as the optical microscope images of the printed droplets. The droplet diameter varied from 250 to 360 μm for the examined laser fluence, while the corresponding droplet volume ranged from 1.15 to 2.9 nL, at 532 nm. Compared to the 355 nm wavelength, the droplet diameter varied from 290 to 410 μm, and the resulting droplet volume ranged from 1.7 to 4.28 nL (Figure 3b).

An important parameter that might affect the viability of printed cells is the velocity (v) of the cell bio-ink [53]. During the droplet formation process, the cell droplet is created due to the expansion of the formed bubble, which is the result of the laser-matter interaction. The rapid expansion of the high-pressure bubble accelerates the forming cell droplet, and the resulting droplet velocity can be high during LIFT-printing [58,59]. Higher laser fluences cause significantly higher droplet accelerations during the droplet formation process and higher droplet decelerations during the droplet landing process [60]. Previous studies have shown that the process-induced high acceleration (or deceleration) and velocity can easily lead to severe cellular injury or death, as observed in the centrifugal force-induced cell damage studies [61,62]. Therefore, the generated jet velocities were investigated for different laser pulse energies and wavelengths. The jet velocity herein was calculated by a linear fit of the jet front distance dependence on the measurement time (Equation (2)). In this study, the initial velocity represented the first instance at which the jet front rose above the donor substrate and was calculated based on the first image frame at which the jet front was visible for the first time after the laser pulse irradiated the donor substrate. On the other hand, the impact velocity represented the velocity at which the jet front reached the receiver substrate and was calculated based on the last frame before the jet landed on the receiver substrate.

**Figure 4:**
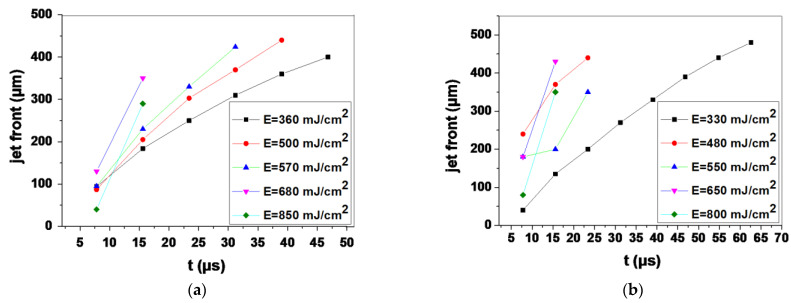
The dependence of the cell suspension jet front position vs. time. (**a**) at 532 nm and (**b**) at 355 nm for various laser fluences.

**Figure 5:**
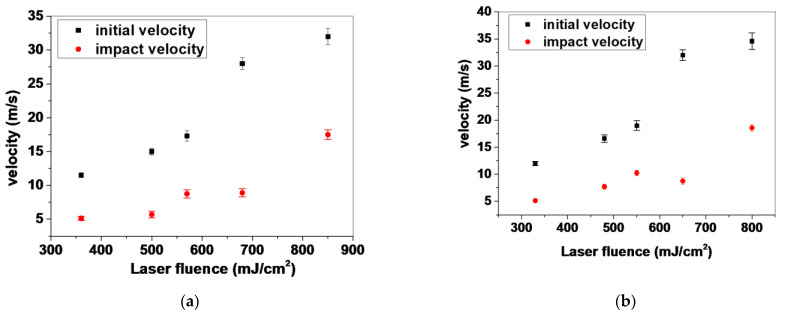
The dependence of produced velocities as a function of laser fluence for both wavelengths. (**a**) at 532 nm and (**b**) at 355 nm for various laser fluences.

An analysis of the front position versus time for the five selected laser fluences is shown in Figure 4. In the case of the 532 nm wavelength, the maximum front velocity ranged from 11.5 m/s to 32 m/s; specifically, 11.5, 15, 17.3, 28 and 32 m/s for jets produced with 360, 500, 570, 680 and 850 mJ/cm^2^ laser fluences, respectively. The slow jets produced at 360 mJ/cm^2^ had a maximum jet front velocity of 11.5 m/s that was reduced to 5.12 m/s upon landing of the droplet. Regarding the laser fluence of 500 mJ/cm2, the produced jet had a maximum ejection velocity of 15 m/s and an impact velocity of 5.76 m/s. In this study, these laser fluences were selected since high-speed experimental results indicated that, in this range of fluences, a stable and reproducible jet is produced with the minimum impact velocity. Compared to UV wavelength, similar laser fluences produced jet with an initial front velocity of 12 m/s, which was reduced to 5.12 m/s and 16.6 m/s (v initial) to 7.7 m/s (v impact), respectively. Figure 5 below shows the initial and impact velocities of the printed droplets for both wavelengths in the laser fluence range between ~350 to ~850 mJ/cm^2^. Since the generated jet velocities were similar for the same laser fluence in both cases (355 and 532 nm), it can be inferred that cell damage became significant above a certain impact velocity threshold and increased as the impact velocity reached higher values, as demonstrated in a previous study [53]. While the factor of jet velocity upon impact plays an important role in post-printing cell viability, the laser wavelength should also be taken into account, as is discussed in Section 3.3.

### 3.2. Cell Growth Kinetics after LIFT

To exclude possible alterations on cell behavior during laser printing, DNA damage experiments after LIFT-printing were conducted for both wavelengths. To evaluate the effect of laser transfer at different wavelengths and selected laser fluences (~350 to 500 mJ/cm^2^) on cell growth, we analyzed the cell number of the printed cell droplets over a period of several days. 

**Figure 6:**
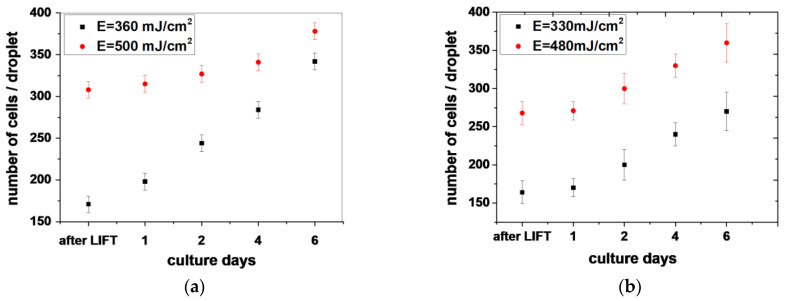
Number of LIFT-printed MDA-MB-468 cells per droplet (**a**) at 532 nm and (**b**) at 355 nm for the indicated laser fluencies. The experiments were conducted at least in triplicates and the number of counted droplets was a minimum of 5 droplets per condition. Error bars indicate SEM.

**Figure 7:**
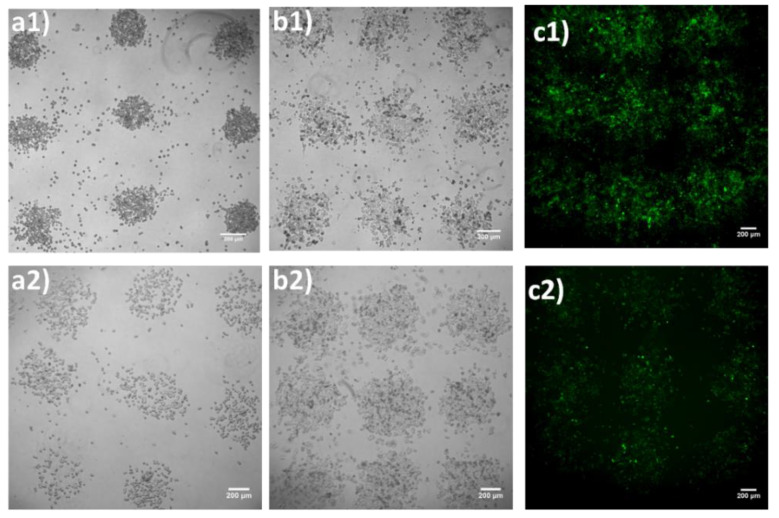
Bright-field (BF) optical microscopy images of LIFT-printed MDA-MB-468-H2B-GFP at 0 h, 4- and 6-days post-printing, at 360 ((**a1**–**c1**) at 532 nm) and 330 mJ/cm^2^ ((**a2**–**c2**) at 355 nm), respectively. Scale bar 200 μm.

At first, optical microscopy images of an array of 6 × 6 droplets were acquired at 30 min, and at 1-, 2-, 4-, and 6-days post-printing, to evaluate the effect of LIFT-printing on cell growth in each droplet, as well as the printing quality. The obtained results are indicated in Figure 6 and Figure 7. Upon printing at 532 nm, we counted 171 ± 10 cells (mean ± SEM) per droplet at 360 mJ/cm^2^, and 308 ± 12 per ejected droplet at a laser fluence of 500 mJ/cm^2^. Similar laser fluences were used with UV wavelength printing (355 nm). The cell numbers were 164 ± 15 and 268 ± 15, for the respective laser fluences. As depicted in Figure 7, 2D cell arrays consisting of MDA-MB-468-H2B-GFP cells were generated and kept in culture for up to six days. The figure indicates that, in all sites where cells were deposited, cell growth was evident. These experiments also demonstrated that the printed cells remained in the areas where they were first printed. For both wavelengths the cells grew in a circular manner, resembling a large colony, continuously proliferating and migrating towards the empty periphery (colony outgrowth) and eventually merging with neighboring spots. Of note, regarding MDA-MB-H2B-GFP cells, the supplementation of the medium with Dox resulted in H2B-GFP expression and detection (through fluorescent microscopy) in less than 24 h. Since only viable cells can respond to Dox, counting of GFP positive cells resulted exclusively in measuring the growth kinetics of living cells (Dox was administered once on the day before the count). In addition, the expression of H2B-GFP upon Dox administration provided strong evidence that the printed cells were not just alive but were also metabolically active.

The presented growth kinetics were slower than those of the same cells in culture. Given the spatial distribution of the printed cells, i.e., confined in a circular area (Figure 7), it was not possible to compare their growth rate with counterparts growing in standard cell culture dishes because the latter were randomly distributed in space and did not slow down due to contact inhibition until they practically reached confluency [63]. Although many cancer cells are in part insensitive to contact inhibition, their growth kinetics are reduced. For this reason, the use of MDA-MB-468-H2B-GFP cultures as controls was not possible. However, the viability of cells, as well as their continuous outgrowth at the periphery of the original printed area for several days, are both clearly depicted in the images of Figure 6.

Although printing had minimal, if any, effect on cell growth, the cells experienced external forces during printing, leading to subcellular alterations which would not necessarily be reflected in cell growth. To address this, the putative LIFT-induced genotoxicity was investigated next.

### 3.3. Quantitative Assessment of LIFT-Induced DNA Damage

There are several potential reasons for cell damage induction during LIFT-printing, including heat and/or shear stress at the point of ejection and light exposure. In the case of UV wavelength, it has been proposed that potential damage could be prevented by use of a thick laser-absorbing layer. Theoretical calculations, based on the penetration depth of the laser-absorbing layer, have shown that cells are exposed to UV irradiation corresponding to energy less than 0.1% of the actual laser beam [10]. 

**Figure 8:**
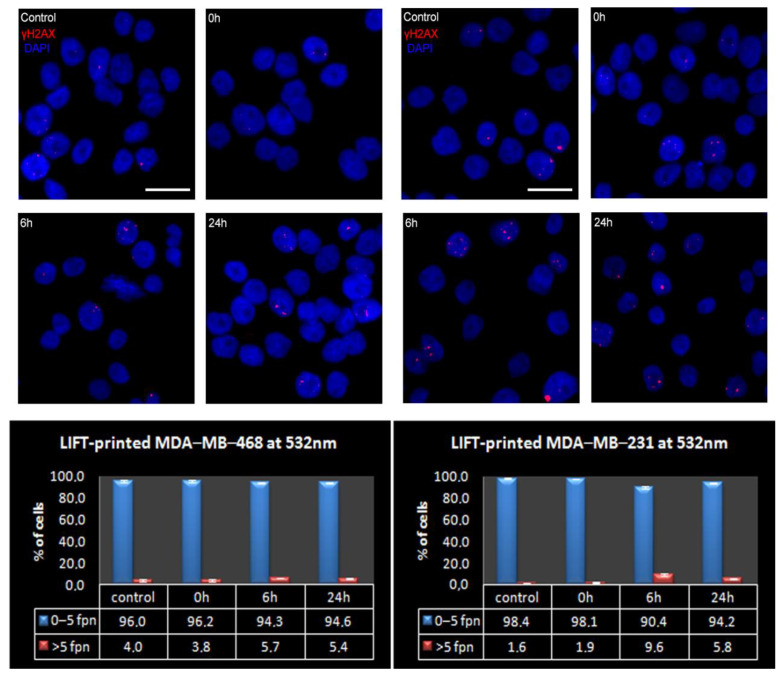
Presence of γH2AX foci on LIFT-printed breast cancer cells at 532 nm (MDA-MB-468 on the left and MDA-MB-231 on the right). (**Top**) Representative IF images of γH2AX foci on control or printed cells at the indicated timepoints. Cell nuclei were counterstained with DAPI, scale bar = 20 μm. (**Bottom**) The percentage of high- and low-DSB-bearing populations at control or given time points after LIFT-printing, fpn-foci per nucleus. The measurements obtained from at least 3 independent experiments and the total cells analyzed were: for MDA-MB-468 control = 1221, 0 h = 1705, 6 h = 1584, 24 h = 1456 and for MDA-MB-231 control = 1609, 0 h = 1896, 6 h = 1769, 24 h = 1746.

In this study, immunostaining against γH2AX was utilized to determine and quantitatively assess whether LIFT-printing induces double-strand breaks (DSBs) in the DNA of printed cells at either wavelength. DSBs, either as direct events or as a consequence of unrepaired single strand breaks during DNA replication, were chosen over other types of DNA damage due to their more severe impact on cells [64,65]. To this end, the endogenous DSB load of MDA-MB-468 cells was initially assessed. Immunofluorescence analysis for γH2AX foci, the most prominent marker of DSBs [66], revealed that less than 1/3 of the exponentially growing MDA-MB-468 cells had zero γH2AX foci in their nuclei (mean 1.5 ± 0.2 and range 0–26 foci per nucleus, n = 1221 cells analyzed). As Table 2 and Figure 8 depict, most cells carried a low DSB load, with >95% of the total population having five γH2AX foci or less per nucleus. Most cell lines were adapted to a high DSB load. For instance, >60% of NIH3T3 cells, a normal-like cell line, bore ≥ 5 foci per nucleus [67]. As cells with five or less foci effectively constituted the entire MDA-MB-468 population, the analysis of LIFT-printed cells was undertaken using the abovementioned ≤ 5 foci per nucleus as the cut-off limit. In more detail, printed MDA-MB-468 cells at 532 nm wavelength and 360 mJ/cm^2^ fluence showed a marginal decrease in the low DSB-bearing population (less than 2%) at 6 h after LIFT, with signs of repair in the consecutive 18 h (Figure 7).

**Figure 9:**
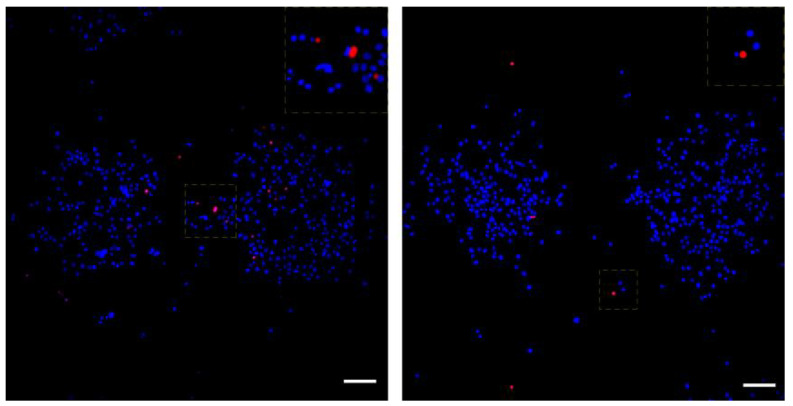
Live-dead assay on printed MDA-MB-468 cells. Representative images of printed cells at 0 h (**left**) and 24 h (**right**). All cells were counterstained with DAPI (blue), while dead cells were also positive for PI (red). The insets on top right are enlargements of the squares in the center of each image with shifted overlay of the red channel (~10 μm) to the right to visualize the DAPI-stained nuclei underneath. Scale bars: 100 μm.

To assess if the minimal but noticeable induction of DSBs in the ~2% of LIFT-printed MDA-MB-468 cells at 532 nm was severe enough to induce apoptosis, we evaluated the kinetics of apoptosis during printing. Since this subpopulation comprised a small fraction of the total printed cells, it was impossible to detect it in our previous cell growth analysis. The printed cells were simultaneously counterstained live with Hoechst 33258 and PI. Hoechst 33258 is cell permeable while PI is not, enabling it to mark apoptotic cells. Our analysis revealed a small induction of apoptosis at 6 h after printing while, remarkably, no extra wave of apoptotic cells was present at 24 h (Figure 9 and Table 3), indicative of negligible threat of LIFT-induced damage to the fate of printed cells.

To strengthen our results, we repeated the same DNA damage analysis at 532 nm, utilizing a second breast cancer cell line, MDA-MB-231, with a notably lower DSB-bearing population [68]. In more detail, the asynchronous cultured MDA-MB-231 cells possessed a 98% low-DSB-bearing population (mean 1.2% ± 0.1 and range 0–25 foci per nucleus, n = 1609 cells analyzed (Table 2)). We assumed that the lower endogenous DSB load of MDA-MB-231 would enable us to detect and more easily assess even smaller changes in the DSB load of the printed cells. Indeed, 6 h after printing, the low-DSB-bearing MDA-MB-231 population decreased by 7.5%, (from 98.1% at 0 h to 90.4% at 6 h); however, 18 h later, more than 50% of this damage was already repaired (94.2% of MDA-MB-231 cells were low-DSB-bearing cells at 24 h, Table 2 and Figure 8). Therefore, even in the case of the more sensitive MDA-MB-231 cells, LIFT-printing was again demonstrated to be a safe method for the precise deposition of cells, even onto stiff substrates in a non-contact manner.

**Figure 10:**
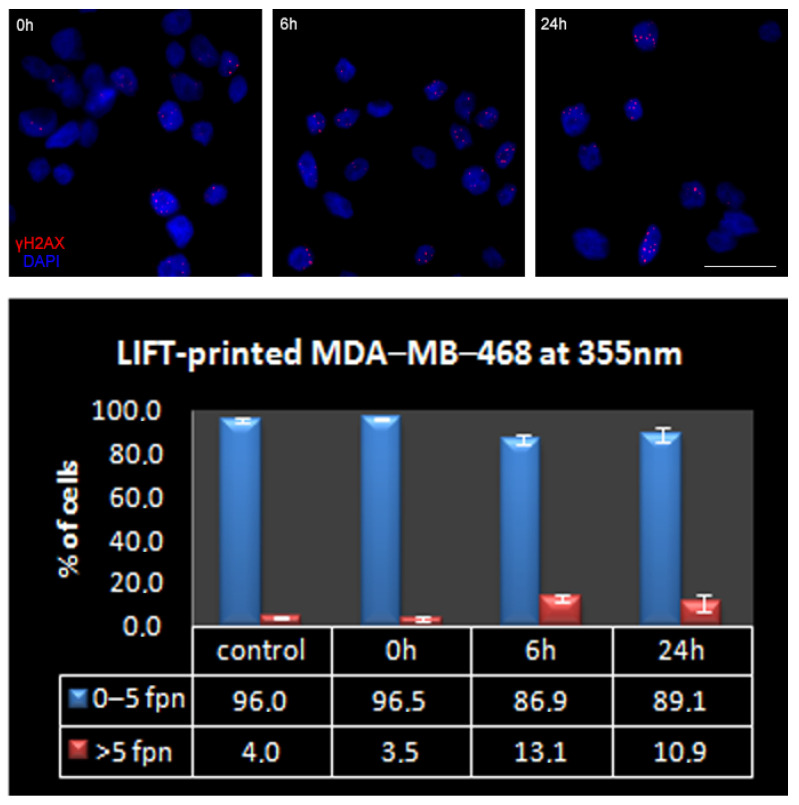
(**Top**) Representative IF images of γH2AX foci of control or printed MDA-MB-468 cells at 355 nm. Cell nuclei were counterstained with DAPI, scale bar = 20 μm. (**Bottom**) The percentage of high- and low-DSB-bearing populations at control or given timepoints after LIFT-printing of MDA-MB-468 at 355 nm, fpn-foci per nucleus. The measurements were obtained from at least 3 independent experiments and the total cells analyzed were: control = 1221, 0 h = 521, 6 h = 1177, 24 h = 1084.

**Figure 11:**
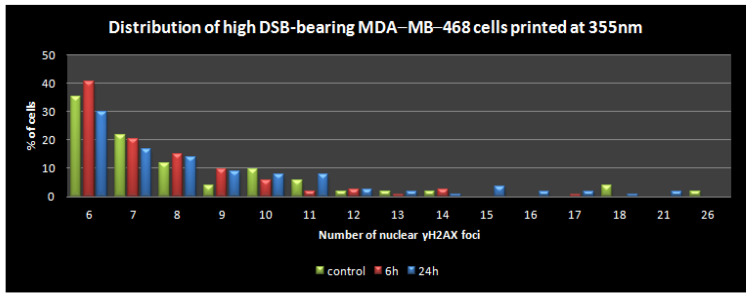
The distribution of high (>5)-DSB-bearing MDA-MB-468 cells in terms of γH2AX foci per nucleus.

Next, we repeated the above assessment with LIFT-printing of MDA-MB-468 cells at 355 nm wavelength, which could be considered more damaging, especially due to the thermal effects of longer exposures (nanosecond laser) [25]. In this case, the low-DSB-bearing population was affected more at 6 h (decreased ~10%), but, again, a substantial recovery in about a quarter of these cells was evident 18 h afterwards (Figure 10). Given the growth rates mentioned earlier (Figure 6 and Figure 7) and the distribution of the high-DSB-bearing population in terms of foci per nucleus (Figure 10 bottom), most of these cells did not suffer gross DSB load that would lead to apoptosis. Interestingly, the repair trend described earlier in the gross analysis of the high-DSB-bearing population at 24 h was more clearly evident in the Figure 11 plot, where the cells with up to 15 γH2AX foci per nucleus were usually decreased at 24 h compared to 6 h, while the remaining population of cells that could be considered potentially pro-apoptotic was practically negligible (<0.1% of the total). Collectively, our results suggest that the induction of DNA damage in cells due to LIFT-printing was marginal. The stress pathways were not investigated since there were no signs of perturbation in the growth or metabolic competence of the printed cells.

Although the use of printing applications is currently favored [25], concerns remain over the safety of bioprinting, and LIFT-printing in particular, with cell-laden bio-inks [69,70]. Our data provide, for the first time, an in-depth analysis of the damaging potential of the LIFT technique by directly measuring the DSBs at two wavelengths (532 and 355 nm). Under the identified optimal LIFT-printing conditions, both wavelengths were able to reproducibly and safely deposit cells on desired surfaces. Although we observed that a wavelength of 355 nm, as compared to 532 nm, may cause some extra damage to the printed cells, our follow-up studies, focusing on repair kinetics and cell growth, clearly indicated that LIFT-printing technologies can be considered a safe application. The current experimental setup, with the simplest cell-laden bio-inks, provides strong evidence for the safety of 2D cell-bioprinting. It is of particular importance to underscore that the tested conditions (low viscosity bio-ink and extremely stiff substrate) offer a putatively harsh environment for the cells. Applications aiming to develop cellularized 3D-printed structures require more viscous bio-inks (usually hydrogels), most often, on less stiff substrates, conditions that are typically less likely to induce damage levels as high as the ones investigated, enabling the extrapolation of our safety evaluation to typical 3D bioprinting modes.

## 4. Conclusions

Bioprinting technologies have unlimited potential in biomedical applications and are reshaping fields such as tissue engineering. Advancements in LIFT techniques are also expected to create major opportunities through their nozzle-free and contactless setup. However, all such technologies must demonstrate their safety compliance to the highest standards if such methodologies are to be introduced to the clinic. Our results show, for the first time at the sub-cellular level, that LIFT-printing of cells is compliant with such standards, producing unperturbed cell growth and negligible genotoxic stress.

## Figures and Tables

**Table 1:** Laser parameters.

Laser	System 1	System 2
Wavelength (nm)	532	355
Pulse duration (ns)	0.6	10
Pulse energy (μJ)	10–18	9–15

**Table 2:** The distribution of breast cancer cells bearing the indicated endogenous γH2AX foci in their nuclei printed at 532 nm wavelength.

Foci Per Nucleus:		0	1	2	3	4	5	>5
**MDA-MB-468**	Mean (%)	30.1	34.9	16.4	7.5	4.9	2.1	4.0
	SEM	3.6	4.8	0.5	1.5	0.8	0.7	1.0
**MDA-MB-231**	Mean (%)	24.9	57.0	10.1	3.7	1.9	0.9	1.6
	SEM	8.9	2.8	3.2	1.9	0.3	0.6	0.5

**Table 3:** The apoptotic fraction of LIFT-printed MDA-MB-468 at 532 nm at the indicated timepoints.

	0 h	6 h	24 h
Measured cells	7758	3739	12,449
Apoptotic cells (mean%)	1.22	1.85	0.81
SEM	0.08	0.15	0.08
